# Technical Feasibility and Safety of Central Venous Ports for Intravenous Chemotherapy in Infants With Retinoblastoma: A Retrospective Study

**DOI:** 10.7759/cureus.52231

**Published:** 2024-01-13

**Authors:** Takatoshi Kubo, Miyuki Sone, Shunsuke Sugawara, Masahiko Kusumoto, Ayumu Arakawa, Chitose Ogawa, Shigenobu Suzuki, Yasuaki Arai, Osamu Abe

**Affiliations:** 1 Radiology, The University of Tokyo Hospital, Tokyo, JPN; 2 Diagnostic Radiology, National Cancer Center Hospital, Tokyo, JPN; 3 Pediatric Oncology, National Cancer Center Hospital, Tokyo, JPN; 4 Ophthalmic Oncology, National Cancer Center Hospital, Tokyo, JPN

**Keywords:** central venous port, chemotherapy, infant, retinoblastoma, vascular access devices

## Abstract

Purpose: The central venous port (CVP) is widely used for intravenous chemotherapy (IVC) in adult patients because of its lower infection rates and easier management than that of a central venous catheter. However, the feasibility and safety of the CVP for IVC in infants remain unknown. This study evaluated the usefulness of CVP for IVC in infants with retinoblastoma.

Methods: The usefulness of CVP was retrospectively evaluated using technical success rates, the safety of CVP placement, and postoperative procedure-related complications in 18 infants with retinoblastoma. This study was conducted at the National Cancer Center Hospital, Chuo-Ku, Tokyo, Japan.

Results: The technical success rate was 100% (18/18) without any procedure-related complications. The sum duration of CVP implantation was 12,836 days (mean: 713 ± 453 days, range: 10-1,639 days). Postoperative complications were observed in two cases; one was a port reversal after 20 days, which was reversed by incisional surgery, and another was a catheter-related bloodstream infection after eight days, resulting in CVP removal. The total incidence of CVP-related infections was 5.6% (1/18) and 0.08/1000 catheter days. No other CVP-related complications were noted.

Conclusion: The use of the CVP for IVC in infants with retinoblastoma was feasible with few complications.

## Introduction

Retinoblastoma is a rare cancer of the infant retina, affecting approximately one in 16,000-18,000 live births annually [1]. Approximately two-thirds of all retinoblastoma cases are diagnosed before the age of two years, with approximately 11% of all cancers occurring within the first year of life [2]. The treatment of retinoblastoma depends on the disease state. Chemotherapy is the treatment of choice, especially for the eye globe salvage [3], and routes of administration include intravenous, intraarterial, intravitreal, and subconjunctival injections. The standard intravenous chemotherapy (IVC) regimen for retinoblastoma is six cycles of vincristine, etoposide, and carboplatin (VEC), although various other regimens have been reported [3].

In IVC for infantile retinoblastoma, anticancer drugs are often administered centrally [4], and a central venous catheter (CVC) is widely used. However, because CVCs have external components, there is a risk of catheter-related bloodstream infections (CRBSIs) [5]. In addition, there are risks of breakage of the external parts of CVCs and accidental removal of CVCs when not in use, burdening the patients and healthcare providers. In clinical practice, CVCs are sometimes inserted and removed during every cycle of chemotherapy because of the difficulty in home management.

A central venous port (CVP) is a central venous infusion system without an exposed external body part and is widely used in adults who require regular central venous drug administration [6]. The risk of infection per 1,000 days of implantation is more than 10 times lower than that of cuffed and tunneled catheters [7], which are considered to have the lowest infection rate among CVCs. In addition, CVPs have no exposed parts outside the body, allowing the patient to lead a normal life when not in use, thereby reducing the burden on the patient. Based on these characteristics, using CVPs for IVC in infants with retinoblastoma, as in adults, may have advantages, such as a lower risk of infection and easier management when not in use. However, there have been no comprehensive reports on CVPs for chemotherapy in infants, and their feasibility and safety remain unknown. Therefore, this study aimed to evaluate the usefulness of a CVP for IVC in infants with retinoblastoma.

## Materials and methods

Study design

This retrospective, observational study was conducted at the National Cancer Center Hospital, Chuo-Ku, Tokyo, Japan, and approved by its Ethics Review Board (approval #2022-110). This study included 18 consecutive CVP implantations in infants performed at our institution between April 2014 and March 2019. Furthermore, medical records were analyzed through March 2021 for the post-CVP implantation status.

Written informed consent was obtained from the parents of all patients prior to the procedure. Due to the retrospective nature of the study, the requirement for additional informed consent for publication was waived, and an opt-out method was used.

CVP placement

As previously reported, 10 experienced interventional radiologists performed all procedures on X-ray fluoroscopy tables equipped with portable ultrasonography (US) during hospitalization [8]. A peripheral intravenous injection route was established before the procedure in all patients. In addition, electrocardiography, pulse oximetry, and non-invasive blood pressure were monitored.

Pediatricians administered thiamylal sodium intravenously (Titosol; Kyorin Pharmaceutical, Tokyo, Japan) at 25-50 mg and ketamine hydrochloride (Ketalar; Daiichi Sankyo, Tokyo, Japan) at a range of 1-2 mg/kg for conscious sedation before and during the procedure. Local anesthesia was achieved with 1% lidocaine before the puncture. No general anesthetic was administered, and endotracheal intubation was not performed.

Under real-time ultrasound guidance with a 10 MHz linear hockey stick probe (Sonosite Titan; Bothell, WA, USA), the indwelling catheters were inserted into the subclavian vein (SCV) with the infraclavicular approach, as previously reported [9]. The puncture was performed with an 18- or 20-gauge needle; however, a 21-gauge micropuncture kit (Cook Medical, IN, USA) was also used appropriately at the operator's discretion. In cases where multiple puncture attempts failed, the access site was changed to the contralateral SCV. After puncturing the SCV, a 5-F Anthron polyurethane catheter (Toray Medical, Tokyo, Japan) was inserted into the superior vena cava with the Seldinger technique using a guidewire and a peel-away sheath. The tip of the indwelling catheter was located at the junction of the right atrium and superior vena cava.

**Figure 1:**
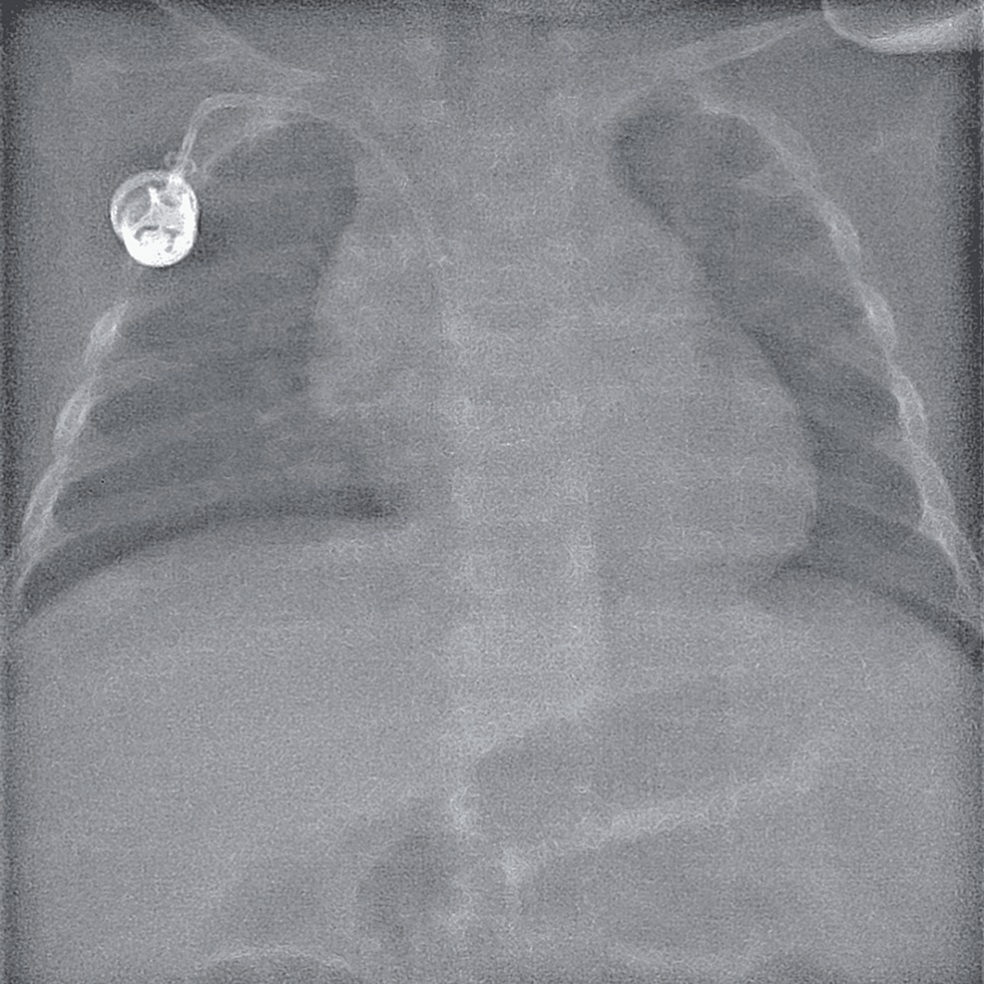
Fluoroscopy after central venous port implantation

A subcutaneous pocket for port implantation was created by blunt dissection of the subcutaneous tissue of the chest wall, approximately 1 cm caudal to the clavicle using a 2-3 cm incision, and then the catheter was connected to the port (CELSITE Babyport: B. Braun Medical Inc., Bethlehem, PA, USA) and implanted in the pocket. The skin incision was sutured with 3-0 absorbable sutures and secured using Steri-Strips (3M, MN, USA) or 2-octyl cyanoacrylate (Dermabond; Ethicon, NJ, USA). After the procedure, the position of the catheter and port system and the presence of pneumothorax were evaluated fluoroscopically (Figure 1).

A 24-gauge Huber point needle was used for puncture when the CVP was used. When the CVP was not used for an extended period, 10 ml of saline was injected at least once every four weeks. Suspected CVP corruption or infection was reported to the interventional radiology department. When CVP infection was suspected or IVC was no longer necessary due to a radical cure, the CVP was removed under local anesthesia.

Measurements

Technical success was defined as the insertion of an indwelling catheter via the SCV and completion of CVP placement. Complications were reviewed during and after CVP placement based on information obtained from medical records. Patients were evaluated separately for procedure-related complications (within 24 hours) and postoperative complications (after 24 hours). Postoperative complications were categorized as either early postoperative complications (24 hours-30 days) or late postoperative complications (>30 days). Furthermore, infection at the port system site or CRBSI was defined as a CVP-related infection. Each patient was followed up until death, CVP removal, loss to follow-up, or the end of the follow-up period. The follow-up was conducted until March 2021.

Statistics

Technical success and complications were evaluated based on the total number of CVP implantations. In addition, as previously reported, CVP-related infections were also assessed for the number of infections per 1000 catheter days [10]. Statistical analysis was performed using the JMP statistical software program (JMP Pro, version 16.2.0; SAS, Cary, North, USA).

## Results

All 18 patients who underwent CVP placement during the study period were included in the analysis. The mean age of the affected children at the time of CVP placement was 6.2 ± 2.9 months (range, 2-11). There were two males and 16 females. All patients underwent CVP implantation for IVC for retinoblastoma, and in all cases, the IVC regimen was VEC. Patient details are shown in Table 1.

**Table 1:** Patient characteristics Except where indicated, data are numbers of patients, with percentages in parentheses. * Data are means ± standard deviations. ** IRCB, International Classification of Retinoblastoma

Parameter	Value
Population	
Age* (range), mo	6.2±2.9 (2–11)
Sex (%)	
Male	2 (11)
Female	16 (89)
Height* (range), cm	66.3±5.4 (58–76)
Weight* (range), kg	8.0±1.3 (5.8–9.96)
Disease status	
Disease distribution (%)	
Unilateral	9 (50)
Bilateral	9 (50)
ICRB** classification (%)	
B	2 (11)
C	7 (39)
D	9 (50)
Cycles of intravenous chemotherapy* (range)	3.1±1.3 (1–6)

There were no deaths, and all patients remained under observation until the end of the study period. The sum duration of CVP implantation in all patients was 12,836 days, with a mean of 713 ± 453 days (range, 10-1,639). Six CVPs were planted at the end of the study period, and the remaining 12 were removed. The CVPs were successfully removed without complications under local anesthesia in all 12 patients who underwent removal during the study period. The mean time from implantation to removal of the 12 patients who underwent removal during the study period was 500 ± 322 days (range, 10-967). Two patients had their CVPs removed in less than 100 days; one in 10 days due to infection and the other in 37 days due to a switch in treatment to intra-arterial chemotherapy. The remaining 10 patients had their CVPs removed after 100 days because of a complete response. 

Technical success was achieved in all cases. The details of the procedures are summarized in Table 2. In five cases, the puncture was difficult to attain, and therefore placement was achieved through the contralateral SCV; the right SCV was used in 12 cases, and the left SCV in six cases. In addition, no procedure-related complications, including arterial bleeding or pneumothorax, occurred. No complications were associated with sedation.

**Table 2:** Details of the central venous port implantation procedure Except where indicated, data are numbers of patients, with percentages in parentheses. * Data are means ± standard deviations.

Parameter	Value
Implantation side (%)	
Right	12 (67)
Left	6 (33)
Procedure time* (range), min	54 ± 24 (25–106)
Radiation exposure time* (range), min	4.3 ± 3.4 (0.9–15.9)
Radiation exposure dose* (range), mGy	6.6 ± 7.6 (0.87–35.2)
Implantation period* (range), days	713 ± 453 (10–1639)

Postoperative complications occurred in two cases: the port system reversal and the CVP-related infection in one case each. All complications were early postoperative complications; there were no late postoperative complications. Port system reversal occurred 20 days after implantation and was difficult to repair manually; therefore, a skin incision was made under local anesthesia, and the port system was repositioned. Re-reversal was not observed after repositioning. A patient with infectious complications developed a fever eight days after CVP placement, and a blood culture demonstrated infection with *Staphylococcus aureus*. Following the associated appearance of purulent drainage from the CVP puncture site, the patient was considered to have a CRBSI. The CVP was removed 10 days after implantation, and the patient was administered antimicrobial agents. After the infection was cured, the CVP was re-implanted. The total incidence of CVP-related infections was 5.6% and 0.08/1000 catheter days. No CVP breakage or venous thromboembolism (VTE) occurred during the observation period. In addition, during the administration of anticancer drugs, there was no accidental disconnection of the access route or subcutaneous leakage of drugs.

## Discussion

This study evaluated the usefulness of CVP for IVC in infants with retinoblastoma based on the technical feasibility of placement and clinical safety.

This study's technical success rate was 100%, and no procedure-related complications were observed. According to the Society of Interventional Radiology Quality Improvement Guidelines for central venous access, the technical success rate of CVP placement via the SCV in adult patients is 95%, with a success rate threshold of 90% [11]. A previous study of CVP placement in children reported success rates as high as 97.1% [12]. The success rate in the present study was similarly high, and CVP placement in infants was considered a technically acceptable procedure.

All patients in this study underwent SCV puncture but not internal jugular vein (IJV) puncture, as commonly used in adult patients. A randomized controlled study of CVC insertion in pediatric cardiac surgery patients reported that the initial puncture success rate was not significantly different between the SCV and IJV; however, the overall success rate was significantly higher for the SCV than for the IJV [13]. In addition, when placing a CVP with an IJV puncture, a subcutaneous tunnel should be formed from the neck to the anterior thoracic region, making the technique more complicated than the SCV puncture. Therefore, SCV puncture might be preferable for CVP placement in infants; however, further studies are needed about the access route.

A randomized controlled trial of central venous access devices for IVC in adults reported a significant reduction in the overall complication rate of approximately 50% with CVPs compared to tunneled CVCs [14], indicating that CVP is safer in terms of post-implantation complications. One case of CRBSI was observed in the present study; however, the number of infections per 1,000 catheter days was 0.08, which is very low, as previously reported for other age groups [12,15]. According to a systematic review of CRBSIs from intravascular devices in adults, the risk of infection per 1,000 days of implantation is significantly lower than that of cuffed and tunneled CVCs [7]. While no studies have directly compared the two in infant cases, the low incidence of CRBSIs in this study suggests that CVP is preferable to CVC in infant IVC in terms of infection risk. No VTE was observed in this study. Meta-analysis of VTE in pediatric cancer patients with the central venous system revealed a VTE incidence of 21% (95% CI:8-37); 20% had tunneled or non-tunneled CVCs, and 12% had implantable ports [16]. Regarding VTE, CVP may be preferable to CVC in pediatric patients with cancer.

From the perspective of daily management, CVPs, which have no exposed external body parts, do not require regular disinfection and protection, have a lower risk of system breakage, and are easier to manage than CVCs. A qualitative study of patient acceptability of central venous access devices for IVC reported that most patients using tunneled CVCs required at least a change in their bathing and sleeping practices [17]. By contrast, the same study reported that patients with CVPs experienced few concerns regarding daily activities. The ease of managing CVP at home, especially by allowing infants to be bathed as usual and kept clean, is considered an advantage for infants and their parents. All patients in this study remained at home without problems between IVC cycles.

By contrast, CVP requires an incision under local anesthesia for removal. In cases where long-term implantation is not expected, CVC is preferable from the perspective of the burden on the patient. In one case in this study, IVC was terminated after one cycle owing to a change in the treatment plan, and possibly, the use of CVC (instead of CVP) would have been less burdensome for the patient. In this study, all patients who had their CVP removed had it removed without any problems. On the contrary, it has been reported that removal becomes difficult after more than 20 months after implantation in children [18], suggesting that it would be best to remove the CVP as soon as possible when it is no longer required.

This study had some limitations. First, this was a single-arm retrospective study; therefore, a direct comparison with CVC was not performed. In addition, because of the small number of patients, infrequent severe complications may not have been evaluated. Furthermore, only retinoblastomas were included in the study; therefore, it is unclear whether using CVP for IVC in infants is safe for other types of cancer. Finally, all the patients in this study received VEC therapy, and it is unclear whether the infection rate would be similar if more myelosuppressive anticancer agents were used. Further studies are required to clarify these limitations.

## Conclusions

The use of CVPs for IVC in infants with retinoblastoma was technically feasible and had a low rate of complications. In terms of daily management, CVPs have advantages for infants and their parents because there are no exposed external body parts requiring routine disinfection and protection. The results of the current study support the use of CVPs for IVC in infants with retinoblastoma.
